# New-onset and relapsed Graves’ disease following COVID-19 vaccination: a comprehensive review of reported cases

**DOI:** 10.1186/s40001-023-01210-7

**Published:** 2023-07-13

**Authors:** Kan Chen, Yiyang Gao, Jing Li

**Affiliations:** Department of Endocrinology and Metabolism, The Institute of Endocrinology, NHC Key Laboratory of Diagnosis and Treatment of Thyroid Disease, The First Hospital of China Medical University, Shenyang, Liaoning China

**Keywords:** COVID-19, SARS-CoV-2, Vaccination, Graves’ disease, Hyperthyroidism

## Abstract

Global Coronavir us disease 2019 (COVID-19) vaccination efforts are being intensified to combat the pandemic. As the frequency of immunization against COVID-19 has increased, some adverse effects related to vaccination have emerged. Within this context, this article reviewed 62 Graves’ disease (GD) cases following COVID-19 vaccination, to probe the potential association between the vaccination and the onset of GD. A comprehensive search of the PubMed, Web of Science, and Scopus databases was conducted to collect GD cases following COVID-19 vaccination up to June 7, 2023. Among the 62 GD cases included in this review, there were 33 (53.2%) new-onset GD and 10 (16.1%) relapsed GD patients following mRNA vaccination, 14 (22.6%) new-onset GD and 4 (6.5%) relapsed GD patients following viral vector vaccination, and 1 (1.6%) relapsed GD patients following inactivated vaccination. Median durations to symptoms onset for new-onset and relapsed GD were 12 (range: 1–60) and 21 (range: 5–30) days following mRNA vaccination, while 7 (range: 1–28) and 14 (range: 10–14) days following viral vector vaccination, respectively. While the definitive pathogenesis of GD following COVID-19 vaccination remains unclear, it might be associated with cross-immune responses triggered by molecular mimicry, and an adjuvant-induced autoimmune/inflammatory syndrome. However, due to the limited number of observed GD cases following COVID-19 vaccination and the lack of systematic experimental studies, a causal relationship between COVID-19 vaccination and the onset of GD has not been definitively confirmed. It should be highlighted that most of GD patients following COVID-19 vaccination experienced positive outcomes after treatment. In the broader context of ending the COVID-19 pandemic and reducing mortality rates, the benefits of COVID-19 vaccination significantly outweigh mild risks such as treatable GD. Adherence to the COVID-19 vaccination schedule is therefore imperative in effectively managing the pandemic.

## Introduction

COVID-19, an acute respiratory infectious disease instigated by severe acute respiratory syndrome coronavirus 2 (SARS-CoV-2) [1], poses a formidable global challenge. As of 7 June 2023, the World Health Organization reported an excess of 700 million COVID-19 cases and over six million associated fatalities all over the world [2]. As the pandemic spreads, researchers have found that SARS-CoV-2 infection can affect not only the respiratory system but also multiple organs, including the endocrine glands [3–6]. At present, the emergence of conditions such as GD, subacute thyroiditis, and diabetes mellitus in association with COVID-19 has been reported [7–14]. In response to this global crisis, COVID-19 vaccination efforts are being intensified globally to control the COVID-19 pandemic. In a meta-analysis, Zheng et al. [15] found that the effectiveness of COVID-19 vaccines against SARS-CoV-2 infection, COVID-19-related hospitalization, intensive care unit hospitalization and death was 89.1%, 97.2%, 97.4%, and 99.0%, respectively, among fully vaccinated populations. Thus, COVID-19 vaccination emerges as a crucial strategy for curtailing SARS-CoV-2 infection and related adverse outcomes,which can provide substantial population protection.

Based on statistics from Our World in Data, as published by the University of Oxford, 70.1% of the global population have received at least a single dose of COVID-19 vaccine, with a total of 13.41 billion doses administered worldwide, and 102,962 doses administered daily [16]. With the rise in COVID-19 vaccination rates, several associated adverse effects have been observed, including fever, fatigue, muscle aches, and some autoimmune diseases [17–22]. Hyperthyroidism refers to thyrotoxicosis caused by the thyroid gland itself producing excessive thyroid hormones, and GD is the most common etiology of hyperthyroidism [23, 24]. GD falls under the umbrella of autoimmune thyroid diseases, characterized primarily by the presence of thyrotropin receptor antibody (TRAb) in the serum [24–26]. While the pathophysiology of GD remains unclear, recent studies suggest that genetic, epigenetic, and environmental factors may all be significant contributors to the disease [27–30]. Recently, there has been a succession of reported cases of GD following COVID-19 vaccination [32–64]. However, it is unclear whether COVID-19 vaccination has any effect on the occurrence and development of GD. This article reviewed 47 new-onset GD cases and 15 relapsed GD cases following COVID-19 vaccination in order to explore whether there is a correlation between COVID-19 vaccination and GD onset and the pathogenic mechanisms.

## Methods

We performed a comprehensive literature review to identify all reports of GD cases following COVID-19 vaccination by searching for indexed articles up to 7 June 2023 in databases including PubMed, Web of Science, and Scopus. Only articles written and published in English were considered. The search strategy employed various keywords both individually and in combination, namely: SARS-CoV-2, COVID-19, thyroid, Graves’ disease, autoimmune thyroid disease, hyperthyroidism, thyrotoxicosis, vaccine, and vaccination. The main inclusion criteria were as follows: 1. case reports, case series, and original articles with adequate case data; 2. letters and comments to the editor published in international journals with adequate case data. The main exclusion criteria were as follows: 1. cases with inadequate data; 2. cases with overlapping patient data; 3. patients previously infected with SARS-CoV-2. From an initial selection of 71 articles, 33 articles met our inclusion criteria, and a total of 62 GD cases following COVID-19 vaccination were included in the analysis.

For each patient, we collected demographic data including sex and age, the type of administered COVID-19 vaccine (mRNA vaccine, viral vector vaccine, inactivated vaccine), the onset time of hyperthyroidism symptoms following COVID-19 vaccination, symptoms and signs at presentation, thyroid function tests (TFTs) such as free thyroxine (fT4), free triiodothyronine (fT3), and thyroid-stimulating hormone (TSH), thyroid antibody tests such as thyroid-stimulating immunoglobulin (TSI), thyroid peroxidase antibodies (TPOAb), and thyroglobulin antibodies (TgAb), as well as other diagnostic examinations such as thyroid ultrasonography and thyroid scintigraphy."

Specific medical therapies and clinical or hormonal follow-up data were also recorded. Based on the 2016 American Thyroid Association guidelines for diagnosis and management of hyperthyroidism and other causes of thyrotoxicosis [24], the diagnostic criteria for GD included: (a) TFTs consistent with hyperthyroidism; (b) positive TRAb or TSI; (c) the presence of a thyroid scan showing high radioactive iodine or ^99m^technetium uptake; or (d) a thyroid ultrasound showing diffuse hypervascularization of the glandular parenchyma.

### New-onset and relapsed GD following COVID-19 vaccination

Among the 62 GD cases following COVID-19 vaccination, the mean age was 42.98 ± 14.34 years, with 45/62 (72.6%) of the patients being female and 17/62 (27.4%) male (Table 2). All patients tested positive for TRAb or TSI. Although GD can affect all ages, it is notably more prevalent among women of reproductive age, and the female-to-male ratio is 5–10: 1 [30, 31]. This gender and age distribution of GD cases following COVID-19 vaccination aligned with that of classical GD cases. The distribution of new-onset and relapsed GD after administration of COVID-19 mRNA vaccines and viral vector vaccines was illustrated in Table 1. However, neither reports have presented new-onset GD cases after receiving inactivated vaccines, nor were there any new-onset and relapsed GD cases after protein subunit vaccine administration. Of the 62 GD patients after receiving COVID-19 vaccines, six female patients and two males were diagnosed with hyperthyroidism through TFTs rather than by presenting hyperthyroidism-related symptoms. Two female patients had received two doses of COVID-19 inactivated vaccines before the mRNA and viral vector vaccines. Excluding the aforementioned 10 patients, the remaining 52 patients developed common hyperthyroidism symptoms after receiving COVID-19 vaccines, such as weight loss, palpitations, and hyperhidrosis, with a median symptom onset time of 12 days. The median durations to symptoms onset for new-onset (12 days *vs* 7 days) and relapsed GD (21 days *vs* 14 days) were longer following COVID-19 mRNA vaccine administration than viral vector vaccination. In female patients, the incidences of both new-onset and relapsed GD following the first dose of COVID-19 vaccines were higher than those after the second dose (Table 2).

**Table 1:** Distribution of the new-onset and relapsed GD after COVID-19 vaccination according to the vaccine type

Vaccine type	New-onset GD			Reference	Relapsed GD			Reference
	No (%)	Age (years, mean ± SD)	Onset time of symptoms (days; Median, min–max)		No (%)	Age (years, mean ± SD)	Onset time of symptoms (days; Median, min–max)	
mRNA vaccine	33/62 (53.2)	42.58 ± 11.97^a^	12 (1–60)^a, b, c^	[32–54]	10/62 (16.1)	43 ± 19.41^a^	21 (5–30)^a, c^	[34, 35, 37, 62]
Viral vector vaccine	14/62 (22.5)	45.79 ± 17.56^a^	7 (1–28)^a, e^	[49, 55–61]	4/62 (6.4)	32.25 ± 9.32^a^	14 (10–14)^a, d^	[57, 63, 64]
Inactivated vaccine	–	–	–	–	1/62 (1.6)	44	7	[34]

^a^ The age was presented as the mean ± SD, while the onset time was expressed as the median (min–max)

^b^ One female patient, who had received two doses of the COVID-19 inactivated vaccine followed by one dose of the mRNA vaccine, developed symptoms two days after mRNA vaccination. That patient was therefore excluded

^c^ Seven patients—five females and two males—were asymptomatic after COVID-19 mRNA vaccination, yet hyperthyroidism was found through routine thyroid function tests. Those patients were therefore excluded

^d^ One female patient, who had received two doses of the COVID-19 inactivated vaccine followed by one dose of the viral vector vaccine, developed symptoms four days after mRNA vaccination. That patient was therefore excluded

^e^ One female patient who remained asymptomatic following the COVID-19 viral vector vaccination was found with hyperthyroidism by routine thyroid function tests. That patient was therefore excluded

The patient with new-onset GD following inactivated vaccine has not been reported as yet

*GD* Graves’ disease, *COVID-19* Coronavirus disease 2019

**Table 2:** Age of subjects and the onset time of GD-related symptoms following COVID-19 vaccination according to the gender of patients

GD	Gender	No	Age (years, mean ± SD)	Number of cases with disease onset after 1st dose (%)	Onset time of symptoms after 1st dose (days; Median, min–max)	Reference	Number of cases with disease onset after 2nd dose (%)	Onset time of symptoms after 2nd dose (days; Median, min–max)	Reference
New-onset GD	F	33	44.55 ± 13.73^a^	18/33 (62.0)^b, c, d, f^	7 (1–50)^a, c, d, f^	[32, 34–36, 40, 42, 44, 48, 55, 57, 59, 60]	11/33 (38.0)^b, c, d, f^	13 (1–60)^b, c, d, f^	[32, 35, 41, 43, 45, 53, 54, 57]
	M	14	41.14 ± 13.73^a^	6/12 (50.0)^b, d^	14 (5–21)^a, d^	[46, 50, 51, 58, 60, 61]	6/12 (50.0)^b, d^	12.5 (2–28)^a, d^	[34, 38, 39, 49, 56, 58]
	Total	47	43.53 ± 13.75^a^	24/41 (58.5)^b, c, d, f^	8.5 (1–50)^a, c, d, f^	[32, 34, 35, 40, 42, 44, 46, 48, 50, 51, 55, 58–61]	17/41 (41.5)^b, c, d, f^	13 (1–60)^a, c, d, f^	[32, 34, 35, 38, 39, 41, 43, 45, 49, 53, 54, 56–58]
Relapsed GD	F	12	39.75 ± 17.42^a^	6/8 (75.0)^b, c, e^	8.5 (5–21)^a, d, e, f^	[34, 35, 61, 62]	2/8 (25.0)^b, c, e^	26.5 (25–28)^a, d, e, f^	[35, 37]
	M	3	47.33 ± 12.58^a^	2/3 (66.7)^b^	17.5 (14–21)^a^	[35, 57]	1/3 (33.3)^b^	30	[34]
	Total	15	41.27 ± 16.46^a^	8/11 (72.7)^b, c, e^	12 (5–21)^a, d, e, f^	[34, 35, 57, 62, 63]	3/11 (27.3)^b, c, e^	28 (25–30)^a, d, e, f^	[34, 35, 37]

^a^ The age was presented as the mean ± SD, while the onset time was expressed as the median (min–max)

^b^ The percentages were calculated individually according to gender and the onset time of symptoms

^c^ One female patient, who had received two doses of the COVID-19 inactivated vaccine followed by one dose of the mRNA vaccine, developed symptoms two days after mRNA vaccination. That patient was therefore excluded

^d^ Seven patients—five females and two males—were asymptomatic after COVID-19 mRNA vaccination, yet hyperthyroidism was found through routine thyroid function tests. Those patients were therefore excluded

^e^ One female patient, who had received two doses of the COVID-19 inactivated vaccine followed by one dose of the viral vector vaccine, developed symptoms four days after mRNA vaccination. That patient was therefore excluded

^f^ One female patient who remained asymptomatic following the COVID-19 viral vector vaccination was found with hyperthyroidism by routine thyroid function tests. That patient was therefore excluded

*GD* Graves’ disease, *COVID-19* Coronavirus disease 2019, *F* female, *M* male

The main characteristics of the 47 new-onset GD patients associated with COVID-19 vaccination were summarized in Table 3. In the reported new-onset GD cases, the main clinical manifestations, thyroid functions, and thyroid ultrasonography were consistent with those of the classical GD cases (Table 3). Notably, a female patient (Case 5), who was inoculated with two doses of the COVID-19 inactivated vaccine, followed by a dose of the mRNA vaccine, experienced hyperthyroidism symptoms, such as palpitations in two days after the mRNA vaccination (Table 3) [33]. Among all the new-onset GD patients following COVID-19 vaccination, 3 female patients (Cases 17, 26, and 47) and 2 males (Cases 15 and 31) did not exhibit hyperthyroidism symptoms after COVID-19 vaccination [37, 39, 47, 49, 52]. Their hyperthyroidism was incidentally identified due to routine TFTs (Table 3). According to literature, most of those patients found symptoms relieved following anti-thyroid drug (ATD) treatment [34, 35, 37–39]. It is worth noting that 3 female patients (Cases 8, 9, and 11) developed hyperthyroidism symptoms after the first COVID-19 mRNA vaccine dose and were diagnosed as new-onset GD [35], as another 2 female patients (Cases 42 and 44) after the first COVID-19 viral vector vaccine dose did [60]. These patients had also received their second doses of COVID-19 vaccines when their conditions stabilized post-ATD treatment. Their conditions did not worsen or require adjusting the doses of ATD and beta-blocker after receiving the second doses [35, 60]. Filippo et al. [65] conducted a case–control study and found that among 64 patients who developed new-onset GD after COVID-19 vaccination, and 20/64 (31.2%) patients experienced post-vaccine early-onset GD (PoVEO) within 4 weeks. The patients with PoVEO GD exhibited unique clinical characteristics as compared to the other 44 patients, including older age at onset, higher male prevalence, and a better initial biochemical and immunologic response to ATD treatments. However, in contrast to those clinical trials, our study is a review of the existing patients, which has aimed to observe the clinical characteristics of new-onset GD cases following COVID-19 vaccination. Based on their clinical characteristics, we analyzed the potential impacts of COVID-19 vaccines on the development of GD. The limited series suggest that for new-onset GD patients following the first COVID-19 mRNA and viral vector vaccine injections, a second dose may be administered once the hyperthyroid conditions are adequately controlled. Nonetheless, constant monitoring of thyroid functions should be kept after the second dose administration.

**Table 3:** Characteristics of cases presenting with new-onset GD following COVID-19 vaccination

Case no	Gender	Age (years)	Vaccine type	Dose	Onset time of symptoms (days)	Symptoms	TFTs	Thyroid antibody tests	Thyroid ultrasound	Thyroid scintigraphy	Medication	Follow up	Reference
1	F	71	mRNA vaccine	2nd	60	Weight loss, asthenia, and atrial fibrillation	fT4 (29.6 pmol/L), TSH (< 0.005 mIU/L)	TRAb (3.6 IU/L), TPOAb (30 IU/mL), TgAb (< 0.9 IU/mL)	Enlarged thyroid and increased vascularity	Diffuse markedly increased uptake over both lobes	Methimazole	TRAb remained positive after 2 months (from 3.6 to 1.9 U/L)	[32]
2	F	42	mRNA vaccine	1st	10–14	Weight loss, asthenia, and palpitations	fT4 (37.32 pmol/L), TSH (< 0.005 mIU/L)	TRAb (4.39 IU/L), TPOAb (2.5 IU/mL)	Enlarged thyroid and increased vascularity	Diffuse markedly increased uptake over both lobes	Methimazole	TRAb remained positive after 2 months (4.39 to 2.1 U/L)	
3	F	54	mRNA vaccine	2nd	10–14	Weight loss, asthenia, and palpitations	fT4 (60.49 pmol/L), TSH (< 0.005 mIU/L)	TRAb (5.1 IU/L), TPOAb (30 IU/mL), TgAb (55 IU/mL)	Enlarged thyroid and increased vascularity	N/A	Methimazole	N/A	
4	F	46	mRNA vaccine	1st	50	Weight loss, palpitations, and irritability	fT4 (41.18 pmol/L), TSH (< 0.005 mIU/L)	TRAb (3.1 IU/L), TPOAb (60 IU/mL), TgAb (90 IU/mL)	Enlarged thyroid and increased vascularity	N/A	Methimazole	N/A	
5	F	40	Inactivated vaccine (× 2), mRNA vaccine	After mRNA vaccination	2	Palpitations	fT4 (27.92 pmol/L), fT3 (8.79 pmol/L), TSH (< 0.015 mIU/L)	TRAb (10.3 IU/L), TPOAb (195.7 IU/mL), TgAb (7.1 IU/mL)	Diffuse hyperplasia and increased vascularity	Diffusely increased radiotracer uptake	Methimazole	N/A	[33]
6	F	47	mRNA vaccine	1st	5	Sweating and palpitations	fT4 (42.73 pmol/L), fT3 (16.94 pmol/L), TSH (< 0.01 mIU/L)	TRAb (22.74 IU/L), TPOAb (11.2 IU/mL), TgAb (320 IU/mL)	Diffuse hyperplasia and increased vascularity	N/A	Methimazole and propranolol	The fT4 level decreased to 22.14 pmol/L and fT3 decreased to 6.44 pmol/L after one month	[34]
7	F	46	mRNA vaccine	2nd	21	Weight loss, emotional lability, sweating, and palpitations	fT4 (100 pmol/L), fT3 (38.96 pmol/L), TSH (< 0.01 mIU/L)	TRAb (9.1 IU/L), TPOAb (146 IU/mL), TgAb (334 IU/mL)	Diffuse hyperplasia and increased parenchymal vascularity	N/A	Methimazole	The fT4 level decreased to 25.35 pmol/L and fT3 decreased to 10.41 pmol/L after one month	
8	F	33	mRNA vaccine	1st	7	N/A	fT4 (45 pmol/L), TSH (0.01 mIU/L)	TRAb (7.3 IU/L)	N/A	N/A	Carbimazole and propranolol	The fT4 level returned to normal after 28 days	[35]
9	F	37	mRNA vaccine	1st	7	N/A	fT4 (60 pmol/L), TSH (< 0.01 mIU/L)	TRAb (3.8 IU/L)	N/A	N/A	Carbimazole and propranolol	The fT4 level returned to normal after 32 days	
10	F	37	mRNA vaccine	2nd	21	N/A	fT4 (72 pmol/L), TSH (< 0.01 mIU/L)	TRAb (11.2 IU/L)	N/A	N/A	Carbimazole and propranolol	The fT4 level returned to normal after 53 days	
11	F	34	mRNA vaccine	1st	26	N/A	fT4 (68 pmol/L), fT3 (23 pmol/L), TSH (0.01 mIU/L)	TRAb (32 IU/L)	N/A	N/A	Carbimazole and propranolol	The fT4 and fT3 levels returned to normal after 58 days	
12	F	33	mRNA vaccine	2nd	9	N/A	fT4 (29 pmol/L), TSH (< 0.01 mIU/L)	TRAb (4.6 IU/L)	N/A	N/A	Carbimazole and propranolol	The fT4 level returned to normal after 64 days	
13	F	43	mRNA vaccine	2nd	13	N/A	fT4 (70 pmol/L), fT3 (> 40 pmol/L), TSH (< 0.01 mIU/L)	TRAb (6.2 IU/L)	N/A	N/A	Carbimazole	The fT4 level returned to normal after 29 days and fT3 returned to normal after 57 days	
14	F	45	mRNA vaccine	1st	2	Generalised body aches, fever (38 °C), chest tightness, and palpitation	fT4 (45.1 pmol/L), TSH (< 0.005 mIU/L)	TRAb (5.75 IU/L)	Heterogeneous thyroid gland with increased vascularity, a few sub-centimetre solid and cystic nodules	N/A	Carbimazole	N/A	[36]
15	M	46	mRNA vaccine	1st	15	Asymptomatic (A routine blood test noticed hyperthyroidism.)	fT4 (20.98 pmol/L), fT3 (7.98 pmol/L)	TRAb (2.9 IU/L)	A slightly enlarged thyroid and increased vascularization	A patchy, very inhomogenous ^99m^technetium accumulation and the uptake was normal	N/A	Thyroid function rapidly returned to normal	[37]
16	M	52	mRNA vaccine	2nd	28	Fever (38 °C), weight loss, and asthenia	fT4 (71.56 pmol/L), fT3 (23.1 pmol/L), TSH (< 0.004 mIU/L)	TRAb (6.48 IU/L), TPOAb (21 IU/mL), TgAb (30 IU/mL)	Enlarged thyroid and increased parenchymal vascularity	N/A	Methimazole and atenolol	The symptoms gradually improved and thyroid function returned to normal after treatment	[38]
17	F	63	mRNA vaccine	2nd	15	Asymptomatic (A routine blood test noticed hyperthyroidism.)	fT4 (30.9 pmol/L), fT3 (4.6 pmol/L), TSH (0.011 mIU/L)	TRAb (positive), TgAb (positive)	A heterogeneous hypervascular gland along with 2 solid isoechoic nodules measuring 1.4 cm and 2.3 cm	N/A	No medication	The TSH level remained suppressed at 0.01 mIU/L after 6 months	[39]
18	M	30	mRNA vaccine	2nd	28	Irritability, palpitations, tremors, restless sleep, and weight loss	fT4 (22.9 pmol/L), TSH (< 0.005 mIU/L)	TSI (positive)	N/A	N/A	Methimazole and atenolol	The TSH level remained fully suppressed and the fT4 level decreased to 14.9 pmol/L	
19	F	28	mRNA vaccine	1st	3	Anxiety, insomnia, palpitations, and distal tremor	fT4 (23.68 pmol/L), fT3 (14.17 pmol/L), TSH (< 0.001 mIU/L)	TRAb (5.85 IU/L), TPOAb (833 IU/mL), TgAb (33 IU/mL)	N/A	N/A	Thiamazole and propranolol	N/A	[40]
20	F	71	mRNA vaccine	2nd	14	Tachycardia, sweating, shortness of breath, leg swelling, dizziness, fever (37.8 °C), and subsequently developed nausea, diarrhoea, abdominal pain and hand tremors	fT4 (92.66 pmol/L), TSH (< 0.01 mIU/L)	TSI 349% (Normal reference range: < 140% baseline)	A stable multinodular disease	N/A	Methimazole and atenolol	The TSH level remained fully suppressed and the fT4 level decreased to 16.73 pmol/L after one month	[41]
21	F	38	mRNA vaccine	1st	12	Nervousness, insomnia, and sweating	fT4 (25.87 pmol/L), fT3 (11.49 pmol/L), TSH (< 0.008 mIU/L)	TSI (12.54 UI/mL), TPOAb (3303.71 IU/mL), TgAb (36.57 IU/mL)	A diffuse decrease in echogenicity with some echogenic septum and increased vascularity	A diffuse goitre with hyperfunctioning	Methimazole	N/A	[42]
22	F	32	mRNA vaccine	2nd	38	Palpitation	fT4 (66.6 pmol/L), fT3 (30.5 pmol/L), TSH (< 0.02 mIU/L)	TSI 420% (Normal reference range: < 140% baseline), TPOAb (239.2 IU/mL), TgAb (7.2 IU/mL)	A heterogeneous background thyroid echogenicity with increase in vascularity	Diffuse markedly increased uptake over both lobes of thyroid, with associated increased blood flow and increased blood pool	Carbimazole and propranolol	Thyroid function was improved after the treatment	[43]
23	F	64	mRNA vaccine	1st	4	Shortness of breath even on a flat road, and subsequently developed palpitations, worsening respiratory distress, decreased urine output, edema of both lower legs, and fever (38.0 °C)	fT4 (42.73 pmol/L), fT3 (23.2 ng/dL), TSH (< 0.008 mIU/L)	TRAb (33.8 IU/L)	Goiter lesions and an increase in vascularization of the parenchyma	N/A	Thiamazole furosemide, and edoxaban tosilate hydrate	Thyroid function returned to normal after 80 days	[44]
24	F	31	mRNA vaccine	2nd	1	Excessive sweating, diarrhea, and shortness of breath during exertion	fT4 (96.14 pmol/L), fT3 (44.2 pmol/L), TSH (< 0.005 mIU/L)	TRAb (11.9 IU/L), TPOAb (481 IU/mL), TgAb (82 IU/mL)	Diffuse hyperperfusion in the thyroid gland	Diffuse hyperaccumulation in the thyroid gland	Thiamazole	Thyroid function returned to normal after 3 months	[45]
25	M	22	mRNA vaccine	1st	14	Subtle tremors in both upper extremities	fT4 (27.3 pmol/L), fT3 (10.9 pmol/L), TSH (0.02 mIU/L)	TRAb (3.76 IU/L)	Heterogeneity of the thyroid parenchyma associated with a marked, diffuse and bilateral intraparenchymal hypervascularization	An increased uptake with homogeneous distribution of the tracer	Methimazole	The patient developed a mild hypothyroidism 6 weeks later. Thyroid function then progressively improved after a thyroid hormone replacement treatment was added	[46]
26	F	44	mRNA vaccine	1st	0	Asymptomatic	TSH (< 0.01 mIU/L)	TRAb (positive)	Volume was 7.4 mL with hypervascularization in both lobes	N/A	N/A	The patient had a history of Hashimoto's disease and levothyroxine had been reduced to 25 μg, with normal thyroid function	[47]
27	F	43	mRNA vaccine	1st	3	Palpitations, sleep disorders, muscle weakness, and heat intolerance	fT4 (65.96 pmol/L), TSH (< 0.002 mIU/L)	TRAb (3.1 IU/L)	N/A	A normal size of the gland, with uniform increased uptake of isotope	Thiamazol and propranolol	Thyroid function returned to normal after 3 months	[48]
28	M	42	mRNA vaccine	2nd	2	Nausea, significant muscle weakness, shortness of breath on exertion, excessive sweating, headache, and difficulty sleeping at night	fT4 (76.71 pmol/L), TSH (0.015 mIU/L)	TRAb (16.1 IU/L)	A prominent heterogeneous and hyperemic gland	Bilateral avid symmetric radionuclide uptake consistent with a hyperfunctioning gland	Methimazole and propranolol	N/A	[49]
29	M	50	mRNA vaccine	1st	14	Fatigue, palpitations, distal tremor, insomnia, anxiety, nervousness, and irritability	fT4 (25.74 pmol/L), fT3 (16.12 pmol/L), TSH (0.001 mIU/L)	TRAb (5 IU/L)	A diffuse enlargement of the thyroid, associated with hypoechogenicity and increased vascularity	An enlarged gland with diffuse uptake of the radioactive	Methimazole	The signs and symptoms of hyperthyroidism gradually improved and FT3 and FT4 levels returned to normal	[50]
30	M	45	mRNA vaccine	1st	14	Palpitations, hand tremors, and weight loss	fT4 (82.63 pmol/L), fT3 (42.35 pmol/L), TSH (< 0.01 mIU/L)	TRAb (17.5 IU/L)	A diffuse swelling of the thyroid gland and an uneven internal hypoechoic image	N/A	Thiamazole and bisoprolol	Thyroid function returned to normal and TRAb decreased to 6.3 IU/L after one year	[51]
31	M	29	mRNA vaccine	2nd	120	Palpitations and mild heat intolerance	fT4 (24.45 pmol/L), fT3 (7.81 pmol/L), TSH (0.008 mIU/L)	TSI (2.9 IU/L)	Heterogeneous echotexture with diffuse hypervascularity and without nodules	A diffuse increased uptake, slightly more intense by the inferior left lobe	Thiamazole	The patient became asymptomatic after three days. The TSH level was still low and fT3 and fT4 levels returned to normal after six months	[52]
32	F	36	mRNA vaccine	2nd	2	Fever (38.9 °C), headache and palpitations, and cervical pain	fT4 (66.02 pmol/L), fT3 (27.57 pmol/L), TSH (< 0.01 mIU/L)	TRAb (26.6 IU/L)	Slight swelling and a focal hypoechoic area with decreased blood flow	Uptake increased to 2.21%	Thiamazole	Subsequently, the patient became euthyroid	[53]
33	F	31	mRNA vaccine	2nd	2	Palpitations, anxiety, and weight loss	fT4 (27.8 pmol/L), TSH (< 0.003 mIU/L)	TRAb (2.21 IU/L)	An enlarged thyroid gland with increased parenchymal Vascularization, and numerous isoechoic nodules on both lobes	Diffusely increased uptake in the thyroid gland and hypoactive nodules on both lobes	Methimazole and propranolol	The patient’s symptoms improved with treatment	[54]
34	F	59	Viral vector vaccine	1st	14	Dyspnea, dizziness, and palpitations	fT4 (29.34 pmol/L), TSH (< 0.0038 mIU/L)	TRAb (positive), TPOAb (< 3.0 IU/mL), TgAb (1494.78 IU/mL)	N/A	N/A	Carbimazole	N/A	[55]
35	F	44	Viral vector vaccine	1st	4	Tremor, heat intolerance, and weight loss	fT4 (35.26 pmol/L), TSH (< 0.0038 mIU/L)	TRAb (positive), TPOAb (206.64 IU/mL), TgAb (2904.39 IU/mL)	N/A	N/A	Carbimazole	N/A	
36	M	70	Viral vector vaccine	2nd	3	Exertional dyspnea, myalgia, palpitation, and weight loss	fT4 (41.06 pmol/L), fT3 (> 30.8 pmol/L), TSH (< 0.0036 mIU/L)	TRAb (3.23 IU/L)	N/A	N/A	Methimazole	N/A	[56]
37	F	46	Viral vector vaccine	1st	1	Chest pain and dyspnea	fT4 (33.92 pmol/L), TSH (0.001 mIU/L)	TRAb (6.42 IU/L), TPOAb (77.72 IU/mL), TgAb (137.5 IU/mL)	Increased vascularity	N/A	N/A	N/A	[57]
38	F	73	Viral vector vaccine	2nd	14	Weight loss and dyspnea	fT4 (73.8 pmol/L), TSH (< 0.008 mIU/L)	TRAb (6.30 IU/L), TgAb (137.5 IU/mL)	Increased vascularity	N/A	N/A	N/A	
39	M	32	Viral vector vaccine	2nd	4	Anxiety, tachycardia, and palpitations	fT4 (38.1 pmol/L), fT3 (12.17 pmol/L), TSH (0.005 mIU/L)	TRAb (7.98 IU/L)	Gland enlargement with pseudonodules and hypervascularization	N/A	Propylthiouracil	Thyroid function returned to normal and TRAb level halved on 100 mg/d dose of propylthiouracil after three months	[58]
40	M	35	Viral vector vaccine	1st	5	Headache, nausea, asthenia, palpitations, tachycardia, mild eyes-redness, and superior palpebral retraction	fT4 (63.84 pmol/L), TSH (< 0.004 mIU/L)	TRAb (3.2 IU/L)	Gland enlargement and hypervascularization	N/A	Propranolol and thiamazole	Thyroid function and TRAb level returned to normal on 5 mg/day dose of thiamazole after three months	
41	F	35	Viral vector vaccine	1st	5	Palpitations, hyperphagia, heat intolerance, and tremor	fT4 (64 pmol/L), fT3 (> 30 pmol/L), TSH (< 0.02 mIU/L)	TSI (24 IU/L), TPOAb (> 1300 IU/mL), TgAb (33 IU/mL)	A diffusely heterogeneous thyroid, with a marked increase in vascularity	N/A	Carbimazole	N/A	[59]
42	M	20	Viral vector vaccine	1st	7	Weight loss, tremors, and bulging of the left eyeball	T4 (18.69 μg/dL), T3 (2.5 ng/mL), TSH (0.002 mIU/L)	TRAb (2.6 IU/L)	N/A	N/A	Carbimazol and propranolol	Thyroid function returned to normal after four months and TED remained stable	[60]
43	F	46	Viral vector vaccine	1st	10	Weight loss and heaviness of both eyes	T4 (21.1 μg/dL), T3 (2.7 ng/mL), TSH (< 0.01 mIU/L)	TRAb (> 40 IU/L), TPOAb (417 IU/mL)	N/A	N/A	Carbimazole and propranolol	Thyroid function returned to normal after two months and TED remained stable	
44	F	19	Viral vector vaccine	1st	28	Hair loss, palpitation, and weight loss	T4 (14.59 μg/dL), T3 (1.62 ng/mL), TSH (< 0.01 mIU/L)	TRAb (7.32 IU/L), TPOAb (703 IU/mL)	N/A	N/A	Carbimazole and propranolol	Symptoms improved and thyroid function returned to normal after two months	
45	F	37	Viral vector vaccine	1st	14	Weight loss, palpitation, and increased frequency of defecation	T4 (17.28 μg/dL), T3 (2.1 ng/mL), TSH (< 0.01 mIU/L)	TRAb (4.37 IU/L), TPOAb (116 IU/mL)	N/A	N/A	Carbimazole and propranolol	Thyroid function returned to normal after three months	
46	M	57	Viral vector vaccine	1st	21	Tremor, palpitations, weight loss, and fatigue	fT4 (51.48 pmol/L), TSH (< 0.005 mIU/L)	TPOAb (positive), TgAb (positive)	A diffusely enlarged gland with increased vascularity	A diffuse goitre with increased uptake	Thiamazole and propranolol	Thyroid function returned to normal after six months	[61]
47	F	68	Viral vector vaccine	1st	30	Asymptomatic	fT4 (46.33 pmol/L), TSH (< 0.01 mIU/L)	TRAb (14.3 IU/L)	Subcentimeter thyroid nodules in the right and left thyroid lobe	Inappropriately normal uptake bilaterally with a region of relative photon deficiency in the right and left thyroid lobes due to nodules	Methimazole, beta-blocker, and apixaban	N/A	[49]

*GD* Graves’ disease, *COVID-19* Coronavirus disease 2019, *F* female, *M* male, *TFTs* thyroid function tests, *T3* triiodo thyronine (Normal reference range: 0.70–2.04 ng/mL), *T4* thyroxine (Normal reference range: 5.74–13.03 μg/dL), *fT4* free thyroxine (Normal reference range: 12–22 pmol/L), *fT3* free triiodothyronine (Normal reference range: 3.1–6.8 pmol/L), *TSH* thyroid-stimulating hormone (Normal reference range: 0.27–4.2 mIU/L), *TRAb* thyroid-stimulating hormone receptor antibodies (Normal reference range: 0–1.5 IU/L), *TPOAb* thyroid peroxidase antibodies (Normal reference range:0–34 IU/mL), *TgAb* thyroglobulin antibodies (Normal reference range: 0–115 IU/mL), *TSI* thyroid-stimulating immunoglobulin (Normal reference range: 0–0.55 IU/L), *TED* thyroid eye disease, *N/A* not available

Table 4 summarized the main characteristics of the 15 relapsed GD cases associated with COVID-19 vaccination. Among those cases, a female patient (Case 14), who had received two doses of inactivated COVID-19 vaccines followed by a viral vector vaccine, developed hyperthyroidism symptoms like palpitations four days after viral vector vaccination (Table 4) [36]. However, among all the relapsed GD patients following COVID-19 vaccination, 3 female patients (Cases 4, 5, and 7) did not present any hyperthyroidism symptoms after COVID-19 vaccination (Table 4) [35]. Their hyperthyroidism was incidentally identified due to routine TFTs. Studies have suggested that approximately 50% of GD patients treated with ATD would experience relapse and it typically occurs between 6 and 18 months after the cessation of ATD treatment [66–68]. Among the 15 cases of relapsed GD after COVID-19 vaccination, a male patient and a female (Cases 1 and 2) relapsed approximately 12 months after ceasing ATD treatment [34]. This potentially represents the natural course of GD. However, in the 3 female patients (Cases 9, 10, and 15), their GD relapse occurred at the 17^th^, 7^th^, and 12^th^ years, respectively, after ceasing ATD treatment [34, 37, 62]. This underlines the necessity for further investigations into the potential correlation between COVID-19 vaccination and GD relapse. Furthermore, a 30-year-old female (Case 14) was diagnosed with GD in October 2018 and has been treated with 2.5 mg of methimazole daily to maintain euthyroidism. She received two doses of COVID-19 inactivated vaccine on April 7 and 26, 2021, and her thyroid function remained normal after COVID-19 vaccination. However, following a dose of the viral vector vaccine on July 19, 2021, she developed symptoms such as palpitations and weight loss four days later. By self-increasing the methimazole dose to 5 mg, she managed to relieve some symptoms, but her fT3 level remained elevated and TRAb was positive one month after adjusting the dose of methimazole [56]. Due to the limited number of reported GD cases following COVID-19 vaccination, and the lack of GD cases after receiving the protein subunit vaccine, it is currently impossible to clarify the causal relationship between COVID-19 vaccination and the onset of GD. Fortunately, most patients who developed GD following COVID-19 vaccination experienced positive outcomes after ATD treatment. In the broader context of ending the COVID-19 pandemic and reducing mortality rates, the benefits of COVID-19 vaccination significantly outweigh mild risks such as treatable GD.

**Table 4:** Characteristics of cases presenting with relapsed GD following COVID-19 vaccination

Case no	Gender	Age (years)	Vaccine type	Dose	Onset time of symptoms (days)	Symptoms	TFTs	Thyroid antibody tests	Thyroid ultrasound	Thyroid scintigraphy	Medication	Follow up	Reference
1	M	49	mRNA vaccine	2nd	30	Palpitations, hand tremors, and sweating	fT4 (49.68 pmol/L), fT3 (20.79 pmol/L), TSH (< 0.01 mIU/L)	TRAb (3.01 IU/L), TPOAb (435 IU/mL), TgAb (236 IU/mL)	A moderate-to-severe increase in parenchymal vascularity of the thyroid gland	N/A	Methimazole and propranolol	The fT4 level decreased to 18.66 pmol/L and fT3 level decreased to 7.87 pmol/L after one month	[34]
2	F	31	mRNA vaccine	1st	21	Hot flushes, weakness, and sweating	fT4 (100.00 pmol/L), fT3 (33.42 pmol/L), TSH (< 0.01 mIU/L)	TRAb (19.30 IU/L), TPOAb (325 IU/mL), TgAb (11 IU/mL)	Moderate increase in parenchymal vascularity of the thyroid gland	N/A	Methimazole and propranolol	The fT4 level decreased to 18.40 pmol/L and fT3 level decreased to 9.53 pmol/L after 5 weeks	
3	M	59	mRNA vaccine	1st	21	N/A	fT4 (49 pmol/L), TSH (< 0.01 mIU/L)	TRAb (12.8 IU/L)	N/A	N/A	Carbimazole	The fT4 level was still elevated	[35]
4	F	74	mRNA vaccine	2nd	11	Asymptomatic (A routine blood test indicating hyperthyroidism.)	fT4 (14 pmol/L), TSH (0.02 mIU/L)	TRAb (6.2 IU/L)	N/A	N/A	Carbimazole	N/A	
5	F	25	mRNA vaccine	2nd	31	Asymptomatic (A routine blood test indicating hyperthyroidism.)	fT4 (15 pmol/L), fT3 (6.3 pmol/L), TSH (0.01 mIU/L)	TRAb (2.9 IU/L)	N/A	N/A	Carbimazole	The fT3 level returned to normal after 123 days	
6	F	41	mRNA vaccine	2nd	28	NA	fT4 (50 pmol/L), TSH (< 0.01 mIU/L)	TRAb (3.9 IU/L)	N/A	N/A	Carbimazole	The fT4 level returned to normal after 31 days	
7	F	24	mRNA vaccine	2nd	63	Asymptomatic (A routine blood test indicating hyperthyroidism.)	fT4 (20 pmol/L), TSH (0.01 mIU/L)	TRAb (2.4 IU/L)	N/A	N/A	NA	The fT4 level returned to normal after 42 days	
8	F	22	mRNA vaccine	1st	5	N/A	fT4 (70 pmol/L), fT3 (> 40 pmol/L), TSH (0.01 mIU/L)	TRAb (5.8 IU/L)	N/A	N/A	Carbimazole and propranolol	The fT4 and fT3 levels returned to normal after 178 days	
9	F	71	mRNA vaccine	2nd	25	Palpitations and sweating	fT4 (45.82 pmol/L), fT3 (17.09 pmol/L)	TRAb (4.2 IU/L)	Multiple confluent anechogenic areas and increased vascularization	Small (partly resected) left lobe and the enlarged right lobe with a patchy inhomogenous tracer distribution and uptake was only mildly increased	N/A	Thyroid function rapidly returned to normal	[37]
10	F	34	mRNA vaccine	1st	5	Swelling of the eyelids, distal tremor, sweating, thermophobia, dyspnoea on exertion, and weight loss	fT4 (32.69 pmol/L), fT3 (22.01 pmol/L), TSH (< 0.01 mIU/L)	TRAb (> 40 IU/L)	N/A	N/A	Thiamazole	N/A	[62]
11	M	34	Viral vector vaccine	1st	14	Weight loss	fT4 (26.61 pmol/L), TSH (< 0.008 mIU/L)	TRAb (4.24 IU/L)	Increased vascularity	N/A	N/A	N/A	[57]
12	F	50	Viral vector vaccine	1st	14	Progressive dyspnoea and palpitations	fT4 (43.76 pmol/L), TSH (0.015 mIU/L)	TRAb (3.77 IU/L), TPOAb (> 1000 IU/mL)	A heterogeneous and hypoechoic thyroid with mildly increased vascularity over the right thyroid lobe	N/A	Propranolol and carbimazole	Thyroid function returned to normal after three months	[63]
13	F	31	Viral vector vaccine	1st	10	palpitations	fT4 (61.39 pmol/L), TSH (0.015 mIU/L)	TRAb (4.13 IU/L), TPOAb (> 1000 IU/mL)	Heterogeneous and hypoechoic echotexture with increased vascularity	N/A	Carbimazole and propranolol	Thyroid function returned to normal after three months	
14	F	30	Inactivated vaccine (× 2) Viral vector vaccine	After Viral vector vaccination	4	Palpitations and weight loss	N/A	N/A	N/A	N/A	Methimazole	Symptoms of thyrotoxicity were improved. Thyroid function remained T3-toxicosis with elevated TRAb one month after methimazole dose adjustment	[64]
15	F	44	Inactivated vaccine	1st	7	Sweating, palpitation, and fatigue	fT4 (34.36 pmol/L), fT3 (14.86 pmol/L), TSH (< 0.01 mIU/L)	TRAb (12.18 IU/L), TPOAb (284 IU/mL), TgAb (119 IU/mL)	Hypoechoic areas separated by fibrous septa and increased parenchymal vascularity	N/A	Methimazole and propranolol	N/A	[34]

*GD* Graves’ disease, *COVID-19* Coronavirus disease 2019, F female, M male, TFTs thyroid function tests, fT4 free thyroxine (Normal reference range: 9.01 ~ 19.05 pmol/L), *fT4* free thyroxine (Normal reference range: 12–22 pmol/L), *fT3* free triiodothyronine (Normal reference range: 3.1–6.8 pmol/L), *TSH* thyroid-stimulating hormone (Normal reference range: 0.27–4.2 mIU/L), *TRAb* thyroid-stimulating hormone receptor antibodies (Normal reference range: 0–1.5 IU/L), *TPOAb* thyroid peroxidase antibodies (Normal reference range:0–34 IU/mL), *TgAb* thyroglobulin antibodies (Normal reference range: 0–115 IU/mL), *N/A* not available

### Possible pathogenesis of COVID-19 vaccination-related GD

Currently, the pathogenic mechanisms of COVID-19 vaccination-related GD has not yet been fully elucidated. Several studies suggest that new-onset and relapsed GD following COVID-19 vaccination may be linked to several potential mechanisms as follows [32–40, 56, 57, 62]. One possible mechanism could be molecular mimicry. The antigen structure of the COVID-19 vaccine closely resembles some autoantigens, eliciting an immune response that could erroneously target host cells with similar autoantigens [32–36, 38, 39, 57]. There are four genera of the coronavirus family: α, β, γ, and δ coronaviruses [69, 70]. SARS-CoV-2, the causative agent of COVID-19, is a member of the Coronaviridae family. This virus is enveloped and possesses a positive single-stranded RNA genome [71–73]. The genome's length varies between 26 and 32 kb, including 6 to 11 open reading frames [74]. It encodes four essential structural proteins—spike, envelope, membrane, and nucleocapsid, all of which play critical roles in the assembly and infectivity of SARS-CoV-2 [69, 75, 76]. Among these proteins, the spike protein is particularly important in facilitating the virus's pathogenicity [77, 78]. It operates as a trimeric fusion type I glycoprotein, binding to the angiotensin converting enzyme 2 (ACE2) on the host cell membrane through its receptor-binding domain (RBD) on the S1 subunit [79]. The binding of the RBD to the ACE2 receptor on the host cell membrane instigates the shedding of the S1 subunit. This event prompts the S2 subunit to adopt a stable post-fusion conformation. The consequent structural changes within the S2 subunit facilitate the integration of the viral lipid membrane into the host cell membrane. This action thereby allows the viral RNA to enter the host cell for replication [80–82]. Hence, due to its crucial role in the infection process, the spike protein is the primary immunogen in the development of the COVID-19 vaccine [83]. Vojdani et al. [84] found that SARS-CoV-2 antibodies could immunoreact with multiple tissue antigens, including those of the thyroid gland. They employed the Basic Local Alignment Search Tool provided by the National Institutes of Health/National Library of Medicine to conduct a sequence matching analysis. Their findings revealed that the amino acid sequences of the SARS-CoV-2 spike protein, nucleoprotein, and membrane protein share a similarity ranging from 50 to 70% with that of thyroid peroxidase [84]. Given that the spike protein plays a pivotal role as an immunogen in the development of the COVID-19 vaccine [83], there is a possibility that the COVID-19 vaccine could incite an autoimmune response against thyroid tissue through molecular mimicry, thereby contributing to the onset of GD. It is important to note that molecular similarity does not necessarily result in the development of GD. A systematic review, including 48 new-onset and 15 relapsed GD cases following COVID-19 vaccination, proposed that individual susceptibility appears to play a critical role in the development of GD following COVID-19 vaccination, regardless of the underlying pathogenic mechanisms [85]. Therefore, other factors such as genetic susceptibility may also contribute to the development of GD. However, it should be noted that unlike our study, this systematic review did not exclude patients with a history of SARS-CoV-2 infection. In our study, we specifically excluded patients who had previously been infected with SARS-CoV-2 to further eliminate the potential impact of SARS-CoV-2 infection on the pathogenesis of GD.

Moreover, another pathogenesis connected to Autoimmune/Inflammatory Syndrome Induced by Adjuvants (ASIA) has been proposed in the literature [32–40, 56, 57, 62]. The term 'ASIA', first proposed by Shoenfeld et al., describes the aberrant autoimmune response post-exposure to a vaccine adjuvant [86]. Common types include post-vaccination syndrome, Gulf War syndrome, and macrophagic myofasciitis syndrome [87]. According to the diagnostic criteria outlined in Table 5, all 62 patients with new-onset or relapsed GD after COVID-19 vaccination satisfied the diagnosis of ASIA [32–64]. In 2013, Perricone et al. [88] first revealed that adjuvant stimuli, such as aluminum and tetramethylpentadecane, could provoke autoimmunity. For instance, aluminum hydroxide was associated with postinoculation phenomena, and silicone was associated with silicosis. In the cohort of patients with relapsed GD after COVID-19 vaccination, a 44-year-old female (Case 15) stood out. With a medical history of GD spanning 13 years, she was administered the first dose of COVID-19 inactivated vaccine on June 10, 2021. A mere week later, symptoms such as hyperhidrosis, palpitations, and fatigue manifested. Subsequently, she was diagnosed with relapsed GD based on laboratory and imaging tests [34]. Given that the inactivated vaccine employs aluminum hydroxide as the adjuvant [89], her GD relapse might be attributed to ASIA triggered by adjuvant-induced enhancement of immune response. However, Perricone et al. [88] suggested that adjuvant-induced autoimmunity could involve multiple mechanisms, including genetic predisposition. Emerging as one of the most promising vaccine types, the mRNA vaccine has been proven with a robust capacity to induce both cellular and humoral immunity [90–92]. The functionality of the mRNA vaccine necessitates its entry into the cell cytoplasm. In this context, Lipid Nanoparticles (LNPs) play an indispensable role as carriers, encapsulating mRNA molecules and facilitating their targeted delivery to specific cells for subsequent action [93]. Animal experiments by Alameh et al. [92] indicated that LNPs could enhance the responses of follicular helper T cells and germinal center B cells, suggesting the potential contribution of LNPs as adjuvants to ASIA syndrome. Furthermore, polyethylene glycol (PEG), a lipid adjuvant in COVID-19 mRNA vaccines, can stabilize LNPs, and PEG exposure may also provoke an amplified immune response leading to thyroid autoimmunity [40]. PEG reactions have been reported, but this condition is exceedingly rare [94]. Despite these insights, the related mechanisms remain unclear, and await large-scale clinical epidemiological studies and systematic experiments to clarify the relationship between the development of GD and COVID-19 vaccination.

**Table 5:** Suggested criteria for the diagnosis of ASIA

Major Criteria	Minor Criteria
Exposure to an external stimuli (Infection, vaccine, silicone, adjuvant) prior to clinical manifestations	The appearance of autoantibodies or antibodies directed at the suspected adjuvant
The appearance of’typical’ clinical manifestations: Myalgia, Myositis or muscle weakness Arthralgia and/or arthritis Chronic fatigue, un-refreshing sleep or sleep disturbances Neurological manifestations (especially associated with demyelination) Cognitive impairment, memory loss Pyrexia, dry mouth	Other clinical manifestations
Removal of inciting agent induces improvement	Specific HLA (i.e. HLA DRB1, HLA DQB1)
Typical biopsy of involved organs	Evolvement of an autoimmune disease

Reference: Perricone et al. [88]

*ASIA* Autoimmune/inflammatory syndrome induced by adjuvants, *HLA* Human leukocyte antigen

### Limitations

We must acknowledge the limitations of our study. Firstly, due to the limited number of existing papers and clinical cases, our results may not fully represent all instances of GD that could potentially appear after COVID-19 vaccination. Secondly, there are variations in the number of individuals receiving different types of COVID-19 vaccines across different regions, which may lead to an overestimation of GD risks associated with certain types of COVID-19 vaccines. Lastly, the proposed pathogenic mechanisms presented in this review are merely attempts to describe the progression of this immune event. Due to a lack of systematic experimental studies, we cannot establish a clear causal relationship between COVID-19 vaccination and the onset of GD. Despite these limitations, our study still provides preliminary information on GD following COVID-19 vaccination, laying a foundation for further research.

## Conclusion

In summary, the COVID-19 vaccination may promote the onset of GD through pathogenic mechanisms such as cross-immune responses triggered by molecular mimicry and ASIA caused by LNPs with adjuvant activity. However, due to the limited number of observed GD cases following COVID-19 vaccination and the lack of systematic experimental studies, a causal relationship between COVID-19 vaccination and the onset of GD could not be definitively confirmed yet. When compared to the total vaccinated population, the occurrence rates of new-onset and relapsed GD after COVID-19 vaccination were extremely low, and most of those GD patients following COVID-19 vaccination experienced positive outcomes after treatment. Therefore, the extensive benefits of COVID-19 vaccination significantly outweigh mild risks such as treatable GD. For patients who have developed GD following the first dose, and those with a history of GD, the COVID-19 vaccine should be actively administered once their conditions are stabilized after treatment. It should be reminded to immediately seek medical attention and have thyroid functions monitored if symptoms of hyperthyroidism develop after COVID-19 vaccination. Treatment regimen may be adjusted as necessary. Currently, clinical epidemiological studies with large sample sizes and systematic experimental studies are needed to elucidate the correlation between COVID-19 vaccination and GD occurrence, and the underlying pathogenic mechanisms.

## Data Availability

Not applicable.

## Abbreviations

COVID-19: Coronavirus disease 2019
SARS-CoV-2: Severe acute respiratory syndrome coronavirus 2
GD: Graves' disease
fT4: Free thyroxine
fT3: Free triiodothyronine
TSH: Thyroid-stimulating hormone
TRAb: Thyrotropin receptor antibody
TSI: Thyroid-stimulating immunoglobulin
TPOAb: Thyroid peroxidase antibodies
TgAb: Thyroglobulin antibodies
TFTs: Thyroid function tests
T4: Thyroxine
T3: Triiodo thyronine
ATD: Antithyroid drug
TED: Thyroid eye disease
ASIA: Autoimmune/inflammatory syndrome induced by adjuvants
LNPs: Lipid nanoparticles
PEG: Polyethylene glycol
HLA: Human leukocyte antigen
F: Female
M: Male
